# Nutritional therapy for MRSA sepsis complicated with pulmonary embolism in children under limited energy and nutrient conditions: a case report

**DOI:** 10.3389/fnut.2025.1484012

**Published:** 2025-02-19

**Authors:** Lin Kong, Li Xia, Fang Zhou, Tian Tan, Zhongmin Gao, Bo Zhou, Yao Jiang, Chengjun Liu, Dandan Pi

**Affiliations:** ^1^Children‘s Hospital of Chongqing Medical University, Chongqing, China; ^2^Ministry of Education Key Laboratory of Child Development and Disorders, Chongqing, China; ^3^Chongqing Key Laboratory of Child Rare Diseases in Infection and Immunity, Children's Hospital of Chongqing Medical University, Chongqing, China; ^4^Department of Nutrition, Children's Hospital of Chongqing Medical University, Chongqing, China; ^5^National Clinical Research Center for Child Health and Disorders, Children's Hospital of Chongqing Medical University, Chongqing, China; ^6^Chongqing Key Laboratory of Pediatric Metabolism and Inflammatory Diseases, Children's Hospital of Chongqing Medical University, Chongqing, China; ^7^Department of Intensive Care, Children’s Hospital of Chongqing Medical University, Chongqing, China; ^8^Department of Pharmacy, Children’s Hospital of Chongqing Medical University, Chongqing, China

**Keywords:** methicillin-resistant *Staphylococcus aureus*, sepsis, pulmonary embolism, nutritional therapy, children

## Abstract

The nutritional treatment of methicillin-resistant *Staphylococcus aureus* sepsis combined with pulmonary embolism presents considerable challenges due to the risks associated with tube placement, coagulation disorders, severe infections, digestive limitations, and fluid restrictions in pediatric patients. This report discusses the case of an approximately 13-year-old female patient admitted with symptoms of right lower limb pain, fever, and cough accompanied by shortness of breath. The patient was assessed to be at moderate risk of malnutrition. In the early stages of treatment, permissive low-calorie enteral nutrition was administered alongside clinical interventions such as anti-infection therapy, anticoagulation, and empyema drainage. In the later stages, supplementary parenteral nutrition therapy was introduced, with careful monitoring of fluids restrictions, infection control, and coagulation index improvements. The patient’s condition improved significantly, and the wounds on the right chest and back healed well. A retrospective review of the literature over the past decade was conducted using domestic and international databases, alongside an analysis of current guidelines for nutritional support in critically ill children.

## 1 Introduction

Sepsis is a global health priority identified by the World Health Organization due to its significant contribution to morbidity and mortality worldwide (1). Various pathogens can cause sepsis, and methicillin-resistant *Staphylococcus aureus* (MRSA) is a major nosocomial pathogen that has become increasingly invasive and epidemic (2–4). Pulmonary embolism (PE), caused by various emboli obstructing the pulmonary artery system, is another critical condition (5). Although pediatric PE diagnoses are often delayed or missed, their incidence has been increasing over the past 5 years (6), with significant implications for morbidity and mortality (7, 8). The management of pediatric MRSA sepsis complicated by PE is particularly challenging, especially regarding nutritional support. However, literature addressing this specific scenario is limited. This study retrospectively reviews the clinical data and nutritional management of a pediatric patient with MRSA sepsis and PE. This research was approved by the hospital ethics committee [Approval number: (2020) Ethics Review (Research) No. 69].

## 2 Case presentation

A 13-year-old girl was admitted to the hospital with a chief complaint of right lower limb pain lasting 7 days, accompanied by fever, cough, and shortness of breath over the preceding 2 days. Seven days before admission, the right lower limb pain began without any apparent cause and progressively worsened, leading to swelling, restricted movement, and an inability to stand. The patient initially sought treatment at a local township clinic and received one session of acupuncture, which provided minimal relief. Two days before admission, her symptoms worsened, with a recorded maximum temperature of 39°C, intermittent dry cough, shortness of breath, and chest pain. Chest radiography performed at the local clinic revealed increased lung markings bilaterally, with no definitive bony abnormalities in the right hip joint. Further investigations at a traditional Chinese medicine hospital showed deep vein thrombosis (DVT) in the right femoral vein via vascular ultrasound, and chest computed tomography (CT) revealed bilateral pneumonia. Based on these findings, the patient was diagnosed with “DVT of the right lower limb and severe pneumonia.” She was treated empirically with cefoperazone/sulbactam for infection and ambroxol for expectoration for 2 days. However, no significant improvement in symptoms was noted, prompting her to transfer to our emergency department with the diagnosis made. Upon admission, the patient exhibited poor appetite and significant clinical deterioration.

On admission, the child’s clinical status was as follows: Physical examination findings: body temperature (T) of 37°C, respiratory rate of 35 breaths/min, heart rate of 148 beats/min, blood pressure of 106/56 mmHg, and oxygen saturation (SpO_2_) of 82%. The patient weighed 40 kg and had a height of 162 cm. The child exhibited normal physical development and moderate nutritional status, but appeared lethargic, with a poor complexion. No skin rash or visible purulent skin lesions were observed. However, cyanosis and dyspnea were noted. Auscultation revealed asymmetrical breath sounds, with decreased sounds on the right lung compared to the left. Moderate to fine moist rales were present in both lungs. The cardiac and abdominal examinations revealed no abnormalities. Significant swelling, tenderness, and restricted movement were observed in the right lower limb.

**Figure 1:**
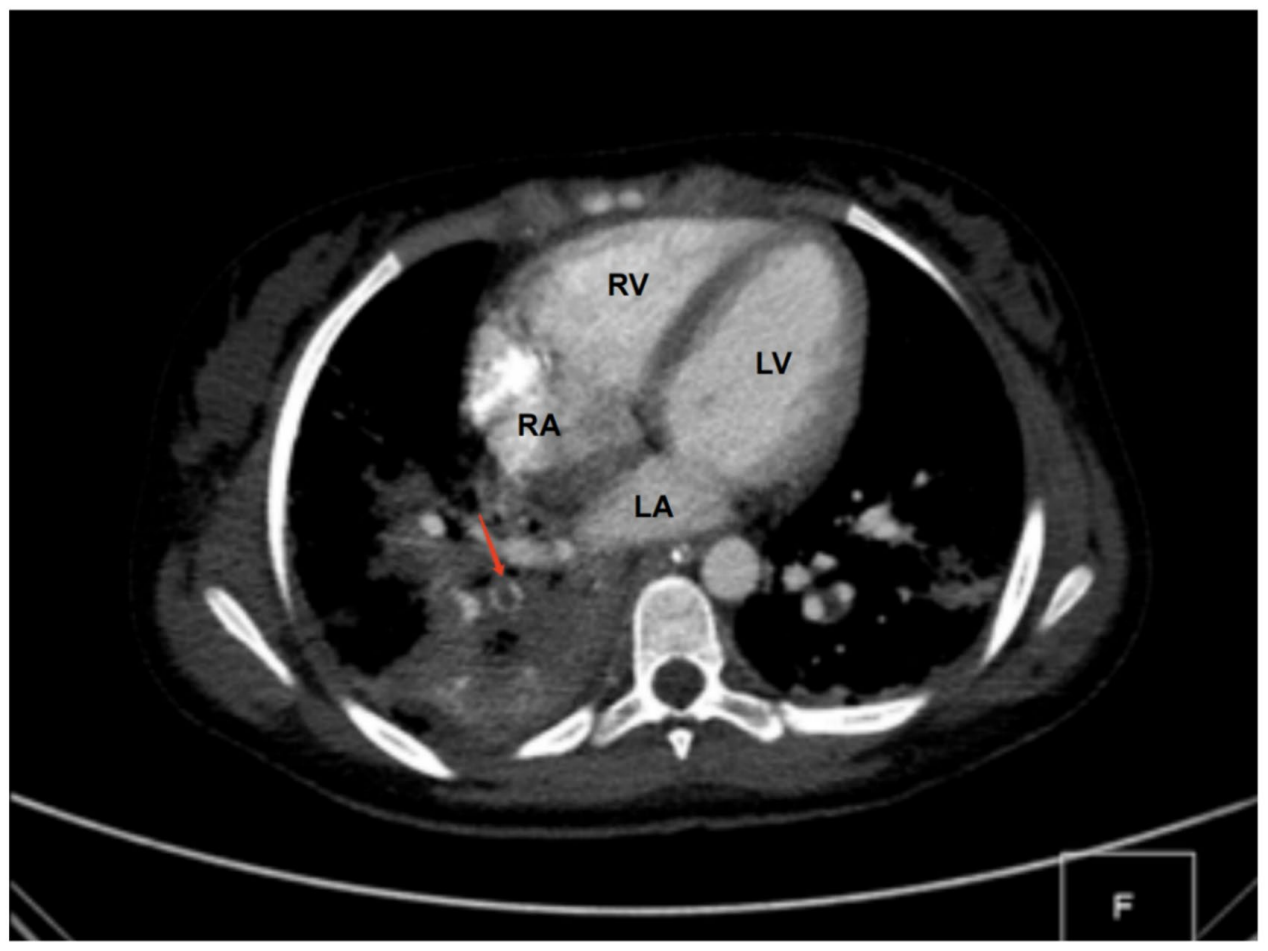
CT findings. A localized filling defect in the middle segment of the posterior basal branch, measuring approximately 4.8 × 5.8 × 4.1 mm (red arrow). LA, left atrium; LV, left ventricle; RA, right atrium; RV, right ventricle.

Diagnostic basis: blood routine test: white blood cell count (WBC) of 11.89 × 10^9^ cells/L (elevated), hemoglobin (HB) level of 86 g/L (decreased), C-reactive protein (CRP) level > 256 mg/L (elevated). Procalcitonin (PCT) level: 42.33 ng/mL (elevated). Coagulation function: Fibrinogen (Fib) level of 6.64 g/L (elevated), prothrombin time (PT) of 14.6 s (prolonged), D-dimer level of 11.94 mg/L (elevated). Liver and kidney functions + electrolytes: total protein (TP) level of 48.9 g/L (decreased), albumin (ALB) level of 23.8 g/L (decreased), phosphorus (P) level of 0.8 mmol/L (decreased), and calcium (Ca) level of 2.13 mmol/L (decreased). Blood culture and sputum (collected from the bronchoalveolar lavage) culture indicated MRSA, but sensitive to vancomycin. On bilateral femoral vein color Doppler ultrasound, thrombus structures were identified in the right popliteal vein, femoral vein, and the initial segment of the right external iliac vein. Color Doppler ultrasound of the right lower limb indicated cellulitis. Chest CT and computed tomography angiography showed multiple consolidations in both lungs with scattered small cavities in the right lower lobe, and bilateral pleural effusion suggestive of infectious lesions. Pulmonary artery reconstruction displayed localized filling defects in the middle segment of the posterior basal branch (approximately 4.8 mm × 5.8 mm × 4.1 mm) (Figure 1). Similar findings were observed in some small arterial branches of the left lower lung. These findings, combined with the clinical history, suggested PE. Cardiac echocardiography revealed left ventricular enlargement, but no significant abnormalities in the posterior wall motion. The left ventricular systolic and diastolic functions were normal.

Primary diagnoses included MRSA sepsis with a pulmonary origin, right femoral vein thrombosis, and PE.

The following clinical treatments were administered: Endotracheal intubation and mechanical ventilation were initiated upon admission. Vancomycin was administered to target MRSA infection. Subcutaneous injections of low-molecular-weight heparin were combined with oral rivaroxaban to manage thrombosis and reduce the risk of embolism. Thoracic drainage surgery was performed to address the empyema. Multiple fiberoptic bronchoscopy lavages were conducted to clear airway obstructions and facilitate lung re-expansion. The patient was placed in lateral and prone positions to improve ventilation and promote lung re-expansion. Specialized care was provided for the surgical wounds to prevent infection and support healing.

## 3 Nutrition management

### 3.1 Nutritional screening and assessment

Nutritional screening was performed using the Screening Tool Risk on Nutritional Status and Growth (STRONGkids) (9), where the patient scored 3 points, indicating a moderate risk of malnutrition. This suggested the need for specialized nutritional intervention. Further evaluation of the patient’s nutritional status revealed a body weight of 40 kg (P25), height of 162 cm (P75–P97), and a body mass index of 15.24 kg/m^2^ (P3–P5) (10). The patient’s food intake had dropped to less than one-third of her pre-illness level, consisting mainly of small amounts of thin porridge. Based on these findings and physical examination, the Pediatric Subjective Global Nutritional Assessment (SGNA) tool was used for a comprehensive evaluation (11). The results confirmed moderate malnutrition.

### 3.2 Estimation of energy requirements

According to the Guidelines for Nutritional Assessment and Supportive Treatment of Critically Ill Children (2018, China, Standard Version), energy requirements were estimated using the Schofield HW formula (12). The formula used was as follows: Energy requirement = Resting energy expenditure × activity factor × stress factor. The activity factor is 1.1 for patients in a bedridden state, and the stress factor is 1.4 for patients in an infected state. The estimated energy requirement was 1983 kcal/day (49.6 kcal/kg/day). The resting energy expenditure alone was calculated as 1,288 kcal/day (32.2 kcal/kg/day).

### 3.3 Nutrition treatment course and nutritional efficacy assessment

**Figure 2:**
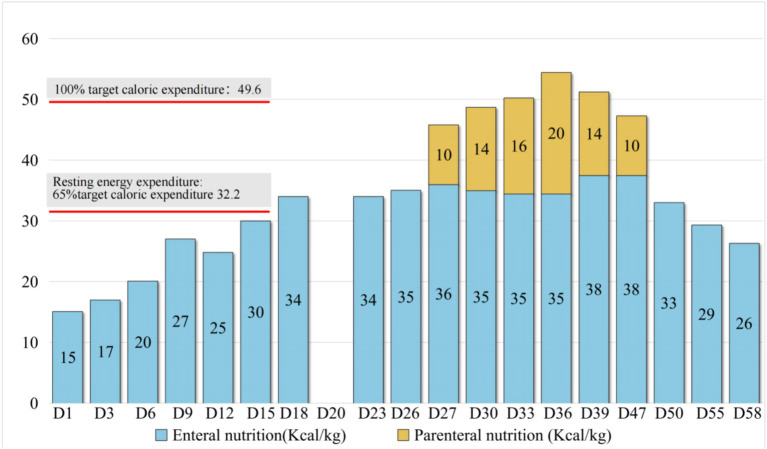
Changes in the energy supply during treatment. Based on the guidelines, venous catheterization risk, fluid management, feeding tolerance, and enteral nutrition intake were gradually increased to the resting energy consumption level during the treatment, approaching 50% of the target energy on day 12 and increasing to 69% of the target energy on day 18. On day 27, supplementary parenteral nutrition support was provided in combination with postoperative wound dehiscence, combined bronchopleural fistula, and clinical nutrition specialist assessment to meet the target energy needs.

**Figure 3:**
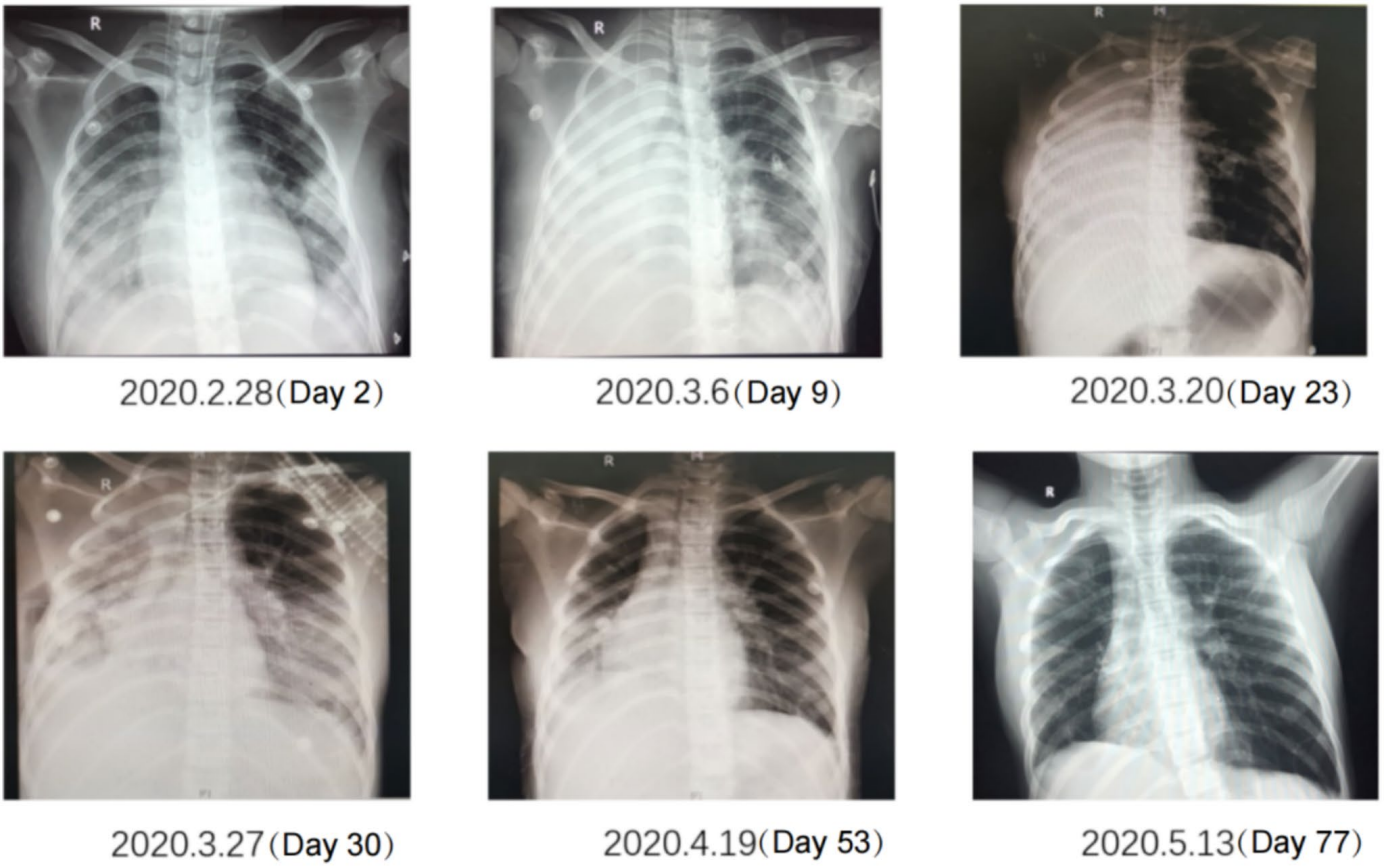
Changes on chest X-ray. Bilateral pneumonia and right pleural effusion gradually improved with treatment progress.

**Figure 4:**
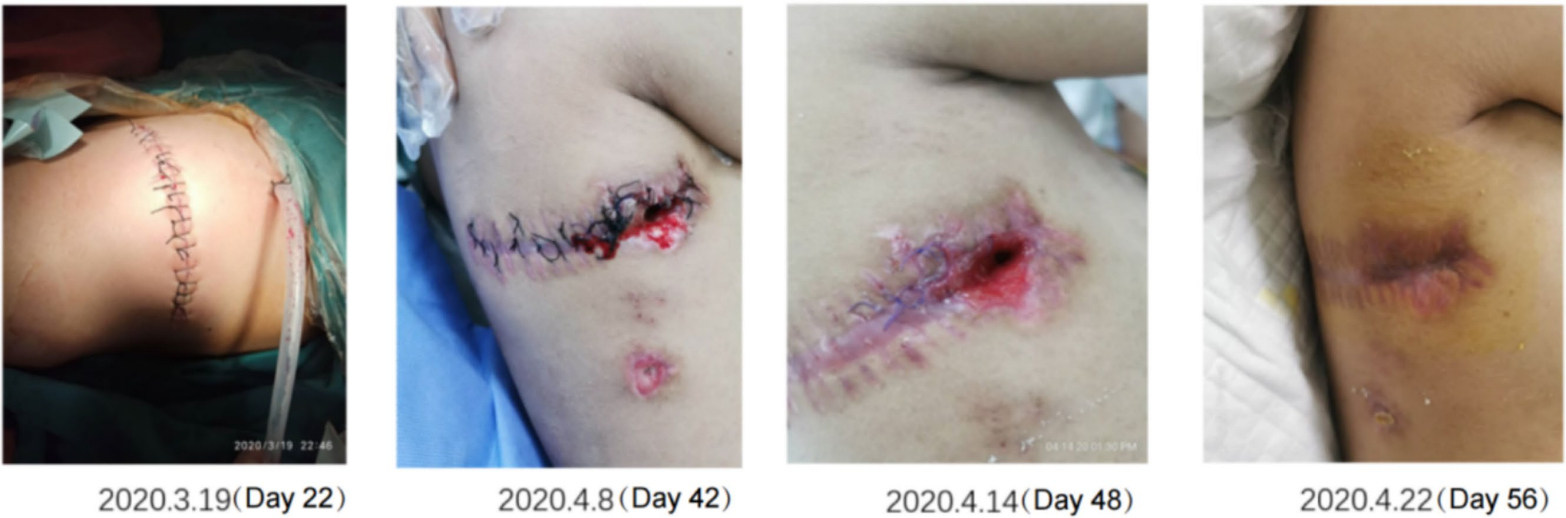
Recovery of the right thoracodorsal wound after drainage. The wound gradually improved and healed as the inflammation was controlled and the nutritional status improved.

Gastrointestinal function assessment: Based on the patient’s medical history, abdominal examination, bedside abdominal X-rays, and abdominal ultrasound, no contraindications to enteral nutrition were identified. Enteral nutrition via a nasogastric tube was initiated. The whole protein formula was administered at 100 mL every 4 h (protein: 0.47 g/kg/day). The feeding volume was gradually increased as tolerated, considering fluid management and tolerance. On the 9th day of admission, the feeding volume was increased to 180 mL (protein: 0.83 g/kg/day). However, the patient developed gastric retention and diarrhea, indicating feeding intolerance. Clinical nutrition consultation recommended switching to a peptide-based formula and adopting intermittent pump feeding (80 mL/h for 2 h, followed by a 2-h break; protein: 0.72 g/kg/day). Feeding intolerance improved, with no signs of reflux or aspiration, and post-pyloric feeding was deemed unnecessary. The feeding rate was gradually increased to 115 mL/h (protein: 1.04 g/kg/day on the 18th day). On the 19th day of admission, the patient underwent thoracic drainage, and pleural and lung biopsies under general anesthesia with endotracheal intubation. On the day of surgery and two days after the surgery, the patient experienced severe infection, renal dysfunction, and low urine output, necessitating suspension of nutritional support fluids. On the 3rd day after surgery (23rd day of admission), once the patient’s condition stabilized, the preoperative enteral nutrition plan was resumed. On the 26th day, peptide pump feeding was increased to 125 mL/h (protein: 1.13 g/kg/day). However, wound dehiscence and a bronchopleural fistula developed. To further improve the nutritional status and promote wound healing, the intensive care unit and clinical nutrition department conducted a multidisciplinary discussion. To address accumulated energy deficits caused by surgical stress, fluid restriction, and feeding intolerance, adjusting the medication fluid volume as much as possible and providing supplementary parenteral nutrition support were recommended. Partial parenteral nutrition was initiated via a PICC line, providing an all-in-one solution containing 19AA amino acids and fish oil-based lipid emulsion. During this period of combined enteral and parenteral nutrition support, total protein intake ranged from 1.33 g/kg/day to 1.95 g/kg/day. Gradually (from day 50 to 58 of admission), before being transferred to a general ward, the patient began consuming an oral whole protein enteral nutrition formula supplemented with a small amount of semi-liquid diet. Changes in enteral and parenteral energy supply during treatment are shown in Figure 2. Changes in the patient’s weight and inflammatory markers throughout the treatment are presented in Table 1. Imaging data and wound healing progression are presented in Figures 3, 4.

**Table 1:** Changes in the nutrition, infection, and coagulation indices during the disease course*.

	D1	D3	D7	D12	D19	D23	D26	D30	D36	D46	D51
Weight (kg)	40	/	39.8	/	/	39.5	/	39.8	40.5	40.8	/
Hemoglobin(g/L)	86	82	63	71	85	79	84	93	86	91	104
Albumin(g/L)	23.8	26.5	31.5	30.9	29	26.3	27.3	26.4	26	26.7	35.4
Platelet count(*10^9^/L)	182	177	440	505	467	437	394	415	492	531	614
PT(s)	13.7	14.5	16.5	20.5	16.1	17.8	15.9	13.3	14	14	/
APTT(s)	32.6	38.5	35.2	39.1	32.8	29.6	27.3	28.6	26.9	28.4	/
WBC(*10^9^/L)	11.98	29.41	23	17.32	29.43	50.19	24.32	20.21	18.21	14.91	16.25
CRP(mg/L)	>256	55	48	96	99	61	90	67	27	18	23
PCT(ng/mL)	42.33	/	2.58	1	0.71	3.53	4.21	1.27	1.49	/	0.49

*Reference range (in our hospital): Hemoglobin: 112–149 g/L; Albumin: 37–52 g/L; Platelet count: 100–472 × 10^9^/L; Prothrombin Time (PT): 9.8–13.3 s; Activated Partial Thromboplastin Time (APTT): 25.0–35.4 s; White blood cells (WBC): 4.4–11.9 × 10^9^/L; C-reactive protein (CRP): <8 mg/L; Procalcitonin (PCT): <0.05 ng/mL.

## 4 Discussion

Severe infections and fluid management are ongoing challenges in the Pediatric Intensive Care Unit. In this study, we reviewed a case of MRSA sepsis complicated by PE, examining the nutritional therapy for such cases to provide reference strategies for the nutritional support of critically ill children.

MRSA is highly pathogenic and can produce various toxins after infection. The spread of infection from the local site to the bloodstream can result in sepsis (13). Furthermore, MRSA infection can lead to changes in coagulation function and immune responses. For patients with severe infections, especially those requiring invasive procedures such as catheterization, the risk of DVT increases, potentially resulting in complications such as PE (14–17). However, reported cases of osteomyelitis and PE caused by *S. aureus* in children remain rare (18, 19). MRSA infection concurrent with PE in pediatric patients is an uncommon clinical presentation, with limited management protocols available. Early diagnosis and intervention are critical. Depending on the severity, options such as anticoagulation, thrombolysis, interventional procedures, or surgery may be necessary (20). For MRSA-induced sepsis, symptomatic supportive treatments such as fluid resuscitation, vasoactive drugs, and anticoagulation therapy are essential based on the patient’s condition (21). Antimicrobial therapy, particularly with vancomycin, remains the first-line treatment for MRSA infections (22, 23). In this case, vancomycin for used for infection control, alongside subcutaneous low-molecular-weight heparin and oral rivaroxaban for anticoagulation therapy upon admission.

Achieving effective blood drug concentrations is crucial for optimal antimicrobial therapy, and nutritional support plays a fundamental role in maximizing the efficacy of antibiotics and other treatments (24, 25). Children with sepsis are often in a high catabolic state during the critical period, influenced by hormonal regulation. Studies have shown that poor baseline nutritional status and inadequate intake after illness can worsen nutritional status, negatively impacting the prognosis of severe sepsis in children (26). Ensuring adequate energy and protein intake, near recommended levels, can improve clinical outcomes in these patients. However, nutritional therapy for children with sepsis presents numerous challenges. First, the body’s stress response can reduce intestinal perfusion, increasing permeability and impairing the intestinal mucosal barrier function, making enteral nutrition therapy more difficult. Research shows that feeding intolerance in septic patients can be as high as 47.7% (27). Second, fluid management and cardiac function maintenance are critical. Fluid restrictions may limit the space available for enteral and parenteral nutrition, requiring a careful balance between fluid intake and nutritional support. At last, the patient presented with DVT and pulmonary thrombosis. While aggressive anticoagulation therapy is necessary, early central venous catheter placement for parenteral nutrition can further increase the risk of thrombosis. Additionally, abnormal coagulation parameters restrict the use of lipid emulsions in parenteral nutrition.

The initiation timing and delivery route for nutritional support remain controversial topics in recent years (28, 29). Studies have demonstrated that even in neonatal and pediatric patients on ECMO, enteral nutrition improves survival rates and reduces complications (30). Similarly, research indicates that most patients requiring noninvasive ventilation can be safely fed enterally after initial ICU stabilization without an increase in respiratory complications (31). Consequently, enteral nutrition is still regarded as the preferred route for nutrient delivery (32). In this case, priority was given to enteral nutrition at the outset, supplemented by short-term parenteral nutrition during the mid-phase due to complications.

Energy supply and nutritional support approach: In the early hospitalization phase, enteral nutrition therapy was initiated cautiously because of clinical interventions, fluid management challenges, and feeding intolerance. Gastric retention and diarrhea observed on the 9th day improved promptly after volume adjustment, formula modification, and a transition to pump feeding. As tolerance improved, post-pyloric feeding was deemed unnecessary. By the 12th day, energy intake reached 50% of the target and increased to 69% by the 18th day. On the 27th day, supplementary parenteral nutrition was introduced, promoted by wound dehiscence, bronchopleural fistula, and a clinical nutrition specialist’s evaluation, Key considerations for this approach were as follows: Current American and European recommendations suggest that energy provision during acute critical illness should not exceed resting energy expenditure. Early parenteral nutrition is generally avoided, with delayed initiation preferred unless enteral nutrition proves inadequate (33). In this case, energy intake gradually increased and reached 50–70% of the target (close to resting energy expenditure) by the second or third week, aligning with guidelines, and allowing parenteral nutrition to be deferred. Guidelines recommend gastric feeding unless aspiration risks or frequent surgical fasting necessitate post-pyloric feeding. This type of feeding is generally implemented only for critically ill children at a high risk of aspiration or those requiring frequent fasting for surgery. Therefore, in this case, although a short period of feeding intolerance was observed in our patient, it improved following adjustment within a short period, and no reflux or aspiration was noted. Therefore, post-pyloric feeding was not implemented in the present case. The present patient had multiple venous thromboses in the right lower limb and PE upon admission. This raised concerns about the increased risk of thrombosis with deep venous catheter placement at this stage. Hence, catheter insertion was delayed to mitigate thrombosis risks. Abnormal coagulation parameters and severe infections warrant careful lipid emulsion use (34). Improved coagulation parameters in conjunction with standardized anticoagulation and antimicrobial therapy later facilitated the safe initiation of supplementary parenteral nutrition.

Protein intake level: Although major nutrition guidelines in China, Europe, and the United States recommend a protein intake of at least 1.5 g/kg for critically ill children (12, 32, 35), achieving this target remains challenging. A United States study of 240 critically ill children reported protein intake as only (40.4 ± 44.2%) of the recommended target in the guidelines (36). In this case, the patient achieved the 1.5 g/kg target only during combined enteral and parenteral nutrition. Protein intake during other phases reached approximately 50–75% of the target, potentially contributing to delayed wound healing.

Selection of enteral nutrition formulas: the initial approach used a polymeric formula, as recommended by the European recommendations for nutrition support in critically ill children (17). When feeding intolerance developed, a pre-digested hydrolyzed formula was introduced. A peptide-based formula with enhanced protein and energy density was later adopted to maximize energy intake, optimize fluid use, and improve tolerance.

Choice of parenteral nutrition formula: Long-term use of pure soybean oil in lipid emulsions is associated with activation of the monocyte–macrophage system, leading to reduced platelet lifespan and increased myeloid cells in the bone marrow (37). Conversely, various lipid emulsions containing fish oil, rich in omega-3 polyunsaturated fatty acids, provide anti-inflammatory and immunomodulatory benefits to critically ill patients (38). Fish oil can also help reduce inflammation-induced damage, decrease energy consumption, and improve nutritional support. Therefore, in this case, a fish oil-containing was chosen, with a cautious dosage of ≤0.5 g/kg to minimize sepsis and thrombosis risks. Glutamine has shown benefits in adult studies involving its intravenous administration in critically ill patients. It improved mortality rates, reduced infection complications, and shortened hospital stay (39). Moreover, glutamine has immunomodulatory properties. However, it was excluded due to its association with significantly increased mortality in pediatric critical care studies (35).

In summary, nutritional therapy for critically ill children with severe infections and pulmonary thrombosis requires a multifaceted approach, encompassing clinical fluid management, risk assessments for catheter placement, coagulation function assessment, infection marker monitoring, and digestive function evaluation. Adjustments to enteral and parenteral nutrition must be phased and flexible, with careful evaluation of the benefits and risks of each approach. The judicious use of immunonutrients, combined with tailored nutritional strategies, is essential for optimizing outcomes in this challenging patient population.

## Data Availability

The original contributions presented in the study are included in the article/supplementary material, further inquiries can be directed to the corresponding author/s.
